# Surgical treatment for synchronous multiple primary lung cancer: Is it possible to achieve both curability and preservation of the pulmonary function?

**DOI:** 10.1111/1759-7714.14164

**Published:** 2021-09-30

**Authors:** Takuya Watanabe, Masayuki Tanahashi, Eriko Suzuki, Naoko Yoshii, Hiroyuki Tsuchida, Shogo Yobita, Kensuke Iguchi, Suiha Uchiyama, Minori Nakamura

**Affiliations:** ^1^ Division of Thoracic Surgery, Respiratory Disease Center Seirei Mikatahara General Hospital Shizuoka Japan

**Keywords:** limited resection, non‐small cell lung cancer, pulmonary function, surgery, synchronous multiple lung cancer

## Abstract

**Background:**

With the advent of high‐resolution chest imaging, the number of patients diagnosed with multiple primary lung cancers is increasing. For the treatment of multiple lung cancers, a surgical procedure that preserves pulmonary function while ensuring curability is required.

**Methods:**

The study population included 85 patients with synchronous multiple primary lung cancer who received surgical resection between January 2010 and September 2020. Patients with synchronous lung cancer within the same lobe were excluded, and only patients with ≥2 involved lobes were included. The postoperative pulmonary function was examined at 3–6 months after the surgery.

**Results:**

Sixty‐seven patients had cancers within the ipsilateral lobe, and 18 patients had cancers in bilateral lobes. Seventy‐six patients (89.4%) underwent combination surgery with limited resection (e.g., segmentectomy and wedge resection). The preoperative pulmonary functions (mean VC/%VC, mean FEV_1_/%FEV_1_, and mean %DLCO) were 3.06 L/100.2%, 2.23 L/96.1%, and 117.2%, respectively, and the postoperative pulmonary functions were 2.45 L/81.4%, 1.87 L/81.2%, and 102.6%. In each parameter, the predicted reductions of pulmonary function were almost the same as the predicted values. The 5‐year survival rate was 85.0%. The 5‐year survival rate according to the most advanced pathological stage was 94.9% for stage I disease, and 62.6% for stage ≥II, which was a significant difference (*p* < 0.001).

**Conclusions:**

Surgical treatment including limited resection, especially segmentectomy and wedge resection, for synchronous multiple primary lung cancer can preserve pulmonary function while ensuring curability.

## Graphical abstract

Our method could adequately predict the postoperative pulmonary function, and the prognosis was good when combined with limited resection.

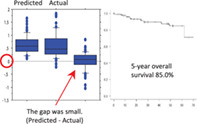

## INTRODUCTION

Recently, with the advent of high‐resolution chest imaging systems and lung cancer screening programs, the number of patients diagnosed with multiple primary lung cancer (MPLC) is increasing.^1^ There are no criteria for the surgical management of MPLC; an appropriate surgical procedure, according to the number and localization of tumors, that preserves pulmonary function while ensuring curability is required. Based on the currently established diagnostic criteria for MPLC,^2^ several studies have shown promising postoperative survival outcomes in MPLC patients,^3^, ^4^, ^5^, ^6^, ^7^, ^8^, ^9^ and the availability of limited resection has been shown.^6^, ^7^, ^8^ However, no studies have previously reported the perioperative pulmonary function in patients who received limited resection for synchronous multiple primary lung cancer (SMPLC). Thus, we aimed to evaluate the benefit of limited resection for SMPLC, including the prognosis and pulmonary function.

## METHODS

### Patients

This retrospective analysis was approved by the Institutional Review Board of Seirei Mikatahara General Hospital (approval number: 19–40, approval date: May 27, 2019). The requirement for informed consent from each patient was waived due to the retrospective nature of the study, with patient information obtained from a database. Among 1792 patients who received surgical resection for primary lung cancer from January 2010 to September 2020, 85 (4.7%) were diagnosed with SMPLC by comprehensive histological assessment according to the diagnostic criteria for MPLC.^2^ By referencing immunohistological staining and cytological/stromal features based on these pathological methods, even in cases with the same histological type, SMPLC and metastatic tumor were able to be distinguished. Patients of SMPLC within the same lobe were excluded because surgical planning was not difficult. Only patients with ≥2 involved lobes were included in this study. In addition, we excluded patients in which one lesion was not indicated for surgical resection due to pulmonary dysfunction, for which nonsurgical interventions (e.g., radiotherapy and simple observation) were performed. Furthermore, in bilateral patients, all lesions had already been confirmed at the primary surgery, so metachronous lung cancers appearing postoperatively were not included.

### Clinical staging methods and principle of lymph node dissection

Thin‐section computed tomography (CT) and 18‐fluorodeoxyglucose positron emission tomography (18‐FDG‐PET) were performed preoperatively, and clinical staging was performed according to the eighth edition of the tumor, node, and metastasis (TNM) classification for lung cancer. In some patients, bronchoscopy, especially endobronchial ultrasound‐guided transbronchial needle aspiration, was used to assess the mediastinal lymph nodes.

For intraoperative lymph node dissection, mediastinal lymph node dissection was performed in lobectomy patients. In segmentectomy patients, the hilar lymph nodes were dissected. Lymph node dissection was not performed in wedge resection patients.

### Examination and calculation of pulmonary function

Pulmonary function testing was performed according to the American Thoracic society.^10^ The patients' pulmonary function (including percentage of vital capacity [%VC], percentage of forced expiratory volume in 1 second [%FEV_1_] and percentage of diffusing capacity of the lung carbon monoxide [%DL_CO_]) was examined pre‐ and postoperatively 6 months after surgery.

The predicted postoperative pulmonary function was calculated as follows. The number of subsegmental bronchi to be resected was counted, and divided by 42 (the total number of subsegmental bronchi). This was taken as the rate of reduction of the pulmonary function. The value obtained by multiplying the preoperative pulmonary function by the reduction rate was calculated as the predicted postoperative pulmonary function.^11^ Wedge resection was considered to be almost equivalent to one subsegmental resection, and was calculated as a 2% reduction of the pulmonary function.

*The calculation formula:*

*x* = *number of subsegmental bronchi to be resected*.

*y* = *number of wedge resections*.

*Predicted reduction volume and rate of pulmonary function (A: without wedge resection, B: including wedge resection)*

$$A:\text{Predicted reduction volume}=\left(\text{preoperative pulmonary function}\right)\left(\frac{x}{42}\right)$$

$$B:\text{Predicted reduction volume}=\left(\text{preoperative pulmonary function}\right)\left(\frac{x}{42}+0.02y\right)$$

*Predicted postoperative pulmonary function (A: without wedge resection, B: including wedge resection)*

$$A:\text{Predicted postoperative pulmonary functio}=\left(\text{Preoperative pulmonary function}\right)\left(\frac{42-x}{42}\right)$$

$$B:\text{Predicted postoperative pulmonary function}=\left(\text{Preoperative pulmonary function}\right)\left(\frac{42-x}{42}-0.02y\right)$$

### Preoperative evaluation and surgical approach

The operative procedure was planned so that the predicted postoperative FEV_1_ was >800 ml, the predicted postoperative %FEV_1_ was >40%, and the predicted postoperative %DL_CO_ was >40%.^12^, ^13^ For lesions with a solid component diameter of >2 cm or lesions diagnosed as clinical stage ≥II due to any lymph node metastasis, lobectomy was selected as much as possible. In addition, when performing two‐stage surgery in bilateral patients, the more‐advanced lesion based on the solid component diameter on preoperative CT and maximum standardized uptake value (SUVmax) on FDG‐PET were operated on first. When the malignancy of multiple lesions was considered to be equivalent, in consideration of the isolated lung ventilation at the second surgery, the one that required less lung resection was operated on first. In bilateral SMPLC, pulmonary ventilation and blood flow scintigraphy were performed before the secondary surgery to calculate the predicted postoperative pulmonary function more precisely.

### Postoperative treatment and follow‐up

According to the most advanced pathological stage, postoperative adjuvant therapy was performed as follows; UFT (a combination of uracil and tegafur) was orally administered for stage I with a tumor invasive diameter of ≥2 cm, and a platinum‐based adjuvant therapy was performed for stage ≥II.

The timing of surveillance for patients who underwent surgical resection of lung cancer in our center was 3 months after surgery, then at 6‐month intervals until 2 years, then annually. Survival was calculated from the date of the second resection to the date of the last follow‐up examination or death.

### Statistical analysis

Fisher's exact test was used to compare categorical variables, while Student's *t*‐test or the Mann–Whitney U test were used for continuous variables. Survival was estimated by the Kaplan–Meier method, and differences in the survival were determined by a log‐rank analysis. *p‐*values of <0.05 were considered statistically significant. All statistical analyses were performed using the StatView software program (SAS Institute Inc.) and EZR (Saitama Medical Center, Jichi Medical University, Saitama, Japan).^14^

## RESULTS

### Patient characteristics

The characteristics of the 85 patients (male, *n* = 40; female, *n* = 45; mean age, 67.8 years [range, 37–84 years]) are shown in Table 1. No patients had a history of pulmonary resection. Sixty‐seven patients had cancers within the ipsilateral lobe, and 18 patients had cancers in the bilateral lobes. The numbers of lesions were as follows: two lesions (*n* = 78), three lesions (*n* = 5), four lesions (*n* = 2). Before the primary surgical intervention, the mean VC was 3.06 L (range, 1.67–4.95 L), the mean %VC was 100.2% (range, 64.9–139.7%), the mean FEV_1_ was 2.23 L (range, 1.20–3.92 L), the mean %FEV_1_ was 96.1% (range, 48.7–158.0%), and the mean %DL_CO_ was 117.2% (range, 50.0–294.5%). There were no significant differences in these variables between ipsilateral and bilateral patients.

**TABLE 1:** Demographic and clinical characteristics of patients with synchronous multiple primary lung cancer

Variables	Total	*N* (%)/mean ± SD (range)	Bilateral	*p* ^a^
		Ipsilateral		
Patients	85 (100%)	67 (78.8%)	18 (21.2%)	
Sex				
Male	40	32	8	0.802
Female	45	35	10	
Age at the primary surgery (years)	67.8 ± 7.60 (37–84)	68.3 ± 6.99 (46–84)	66.2 ± 9.37 (37–80)	0.468
Number of lesions				
2	78 (91.8%)	63 (94.0%)	15 (83.3%)	0.143
≥3	7 (8.2%)	4 (6.0%)	3 (16.7%)	
3	5	4	1	
4	2	0	2	
Preoperative pulmonary function at the primary surgery				
VC (L)	3.06 ± 0.84 (1.67–4.95)	3.11 ± 0.86 (1.67–4.95)	2.90 ± 0.68 (2.00–4.16)	0.312
%VC (%)	100.2 ± 15.1 (64.9–139.7)	101.1 ± 15.6 (64.9–139.7)	97.3 ± 13.5 (70.4–129.3)	0.184
FEV_1_ (L)	2.23 ± 0.62 (1.20–3.92)	2.27 ± 0.87 (1.20–3.92)	2.11 ± 0.69 (1.48–3.22)	0.158
%FEV_1_ (%)	96.1 ± 20.5 (48.7–158.0)	97.3 ± 21.7 (48.7–158.0)	91.1 ± 16.1 (63.0–118.5)	0.124
%DLco (%)	117.2 ± 40.2 (50.0–294.5)	116.0 ± 57.3 (50.0–227.1)	119.9 ± 63.0 (66.1–294.5)	0.958
Preoperative pulmonary function at the secondary surgery				
VC (L)			2.46 ± 0.61 (1.44–3.87)	
%VC (%)			84.4 ± 14.0 (59.4–119.4)	
FEV_1_ (L)			1.80 ± 0.47 (1.20–3.09)	
%FEV_1_ (%)			80.9 ± 16.1 (57.7–115.3)	
%DLco (%)			106.5 ± 55.2 (61.4–246.5)	

Abbreviations: DLco, diffusing capacity of the lung carbon monoxide; FEV_1_, forced expiratory volume in 1 second; SD, standard deviation; VC, vital capacity.

^a^ Comparisons between patients with ipsilateral and bilateral disease.

### Surgical procedure

The details of the surgical procedures of the ipsilateral and bilateral patients are shown in Table 2. In the ipsilateral patients, all underwent one‐stage surgery. All bilateral patients, apart from one patient who underwent one‐stage surgery (*n* = 17) underwent two‐stage surgery. Median interval ± standard deviation (SD) between contralateral surgery 7 ± 13.6 months (range, 2–51 months). Among the 85 patients, nine (10.6%) underwent resection of two lobes (upper‐middle lobectomy [*n* = 5], middle‐lower lobectomy [*n* = 3], middle lobectomy and left lower lobectomy [*n* = 1]). The remaining 76 patients (89.4%) were combined with limited resection (e.g., segmentectomy and wedge resection). No patient underwent pneumonectomy.

**TABLE 2:** Surgical procedures for synchronous multiple primary lung cancer

	N	%
Ipsilateral	67	100.0%
Only lobectomy (L‐L)	8	12.0%
Combined sublobar resection	59	88.0%
L‐S	10	14.9%
L‐W	24	35.8%
S‐S	10	14.9%
S‐W	11	16.4%
W‐W	1	1.5%
S‐S‐W	1	1.5%
L‐W‐W	1	1.5%
S‐W‐W	1	1.5%
Bilateral^a^	18	100.0%
Only lobectomy (L‐L)	1	5.6%
Combined sublobar resection	17	94.4%
L/S	5	27.6%
S/L	3	16.6%
S/S	3	16.6%
W‐L^b^	1	5.6%
W/S	1	5.6%
W/W	1	5.6%
S/L‐W	1	5.6%
L‐W/S‐S	1	5.6%
L‐W/S‐W	1	5.6%

Abbreviations: L, lobectomy; S, segmentectomy; W, wedge resection.

^a^ In bilateral patients, the surgical procedure was shown as follows: primary surgery/secondary surgery.

^b^ One patient received one‐stage surgery.

### Postoperative pulmonary function

**FIGURE 1:**
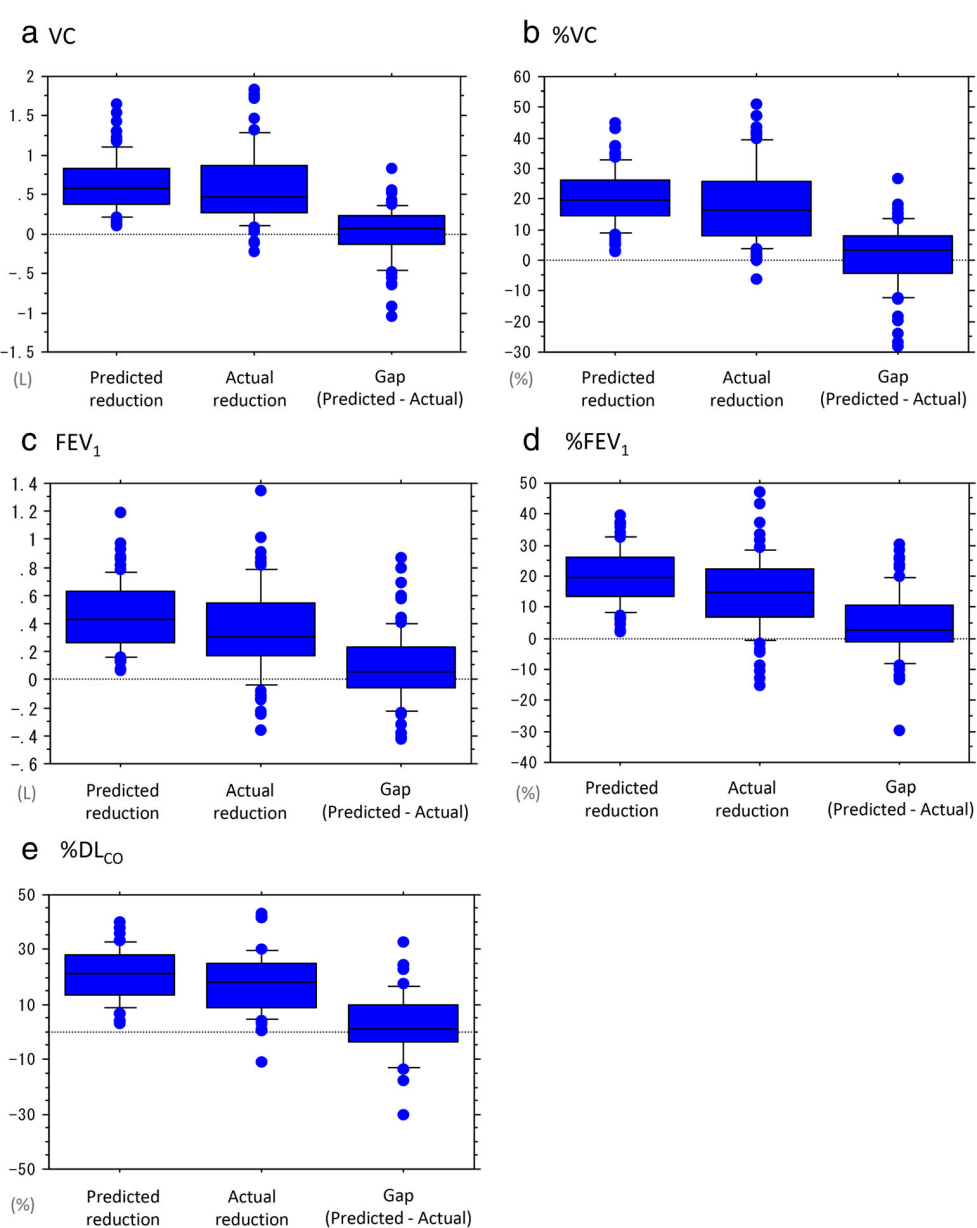
The box‐plot shows that there were no serious discrepancy between the predicted reduction in and actual pulmonary function after surgery for synchronous multiple primary lung cancer ((a) VC, (b) %VC, (c) FEV_1,_ (d) %FEV_1_, (e) %DL_co_)

Postoperative pulmonary function is shown in Table 3, and a box‐plot for each pulmonary function is shown in Figure 1. The predicted volume and percentage reduction after surgery was as follows; mean ± SD and range: VC, 0.63 L ± 0.34 (range, 0.10–1.66 L); %VC, 20.7% ± 9.3 (range, 2.8–45.2%); FEV_1_, 0.45 L ± 0.23 (range, 0.06–1.20 L); %FEV_1_, 19.8 ± 8.9 (range, 2.3–39.5%); %DL_CO_, 20.8 ± 9.0 (range, 3.1–40.1%). In contrast, the actual postoperative pulmonary function (volume and percentage reduction) were as follows; mean ± SD and range: postoperative VC, 2.45 L ± 0.70 (range, 1.12 to 4.29 L); actual reduction of VC, 0.59 L ± 0.46 (range, −0.23 to 1.84 L); postoperative %VC, 81.4% ± 17.4 (range, 43.2 to 124.8%); actual reduction of %VC, 18.6% ± 13.3 (range, −5.9% to 50.9%); postoperative FEV_1_, 1.87 L ± 0.54 (range, 0.81 to 3.43 L); actual reduction of FEV_1_, 0.36 L ± 0.31 (range, −0.36 to 1.35 L); postoperative %FEV_1_, 81.2% ± 20.0 (range, 34.6% to 144.4%); actual reduction of %FEV_1_, 14.6% ± 12.3 (range, −15.2% to 47.0%); postoperative %DL_CO_, 103.1% ± 38.5 (range, 52.6% to 226.4%), actual reduction of %DL_CO_, 19.7% ± 18.3 (range, −11.0% to 121.4%). Negative numbers indicate that the postoperative lung capacity was higher than the preoperative lung capacity.

**TABLE 3:** Predicted and actual volume reduction

Variables	Mean ± SD [range]			*p* ^b^
	Total	Ipsilateral	Bilateral^a^	
Predicted reduction in pulmonary function				
VC (L)	0.63 ± 0.34 [0.10–1.66]	0.64 ± 0.35 [0.10–1.66]	0.61 ± 0.32 [0.12–1.31]	0.869
%VC (%)	20.7 ± 9.3 [2.8–45.2]	20.5 ± 9.2 [3.1–45.2]	21.3 ± 10.2 [2.8–43.1]	0.778
FEV_1_ (L)	0.45 ± 0.23 [0.06–1.20]	0.46 ± 0.24 [0.06–1.20]	0.43 ± 0.20 [0.08–0.70]	0.855
%FEV_1_ (%)	19.8 ± 8.9 [2.3–39.5]	19.8 ± 8.9 [2.6–37.6]	19.8 ± 9.5 [2.3–39.5]	0.993
%DLco (%)	20.8 ± 9.0 [3.1–40.1]	20.4 ± 8.9 [4.0–40.1]	22.1 ± 8.1 [3.1–33.3]	0.327
Actual reduction in postoperative pulmonary function				
VC (L)	0.59 ± 0.46 [(−0.23)‐1.84]	0.61 ± 0.50 [(−0.23)‐1.84]	0.53 ± 0.28 [0.14–1.17]	0.881
%VC (%)	18.6 ± 13.3 [(−5.9)‐50.9]	18.6 ± 13.5 [(−5.9)‐50.9]	18.6 ± 13.0 [4.0–47.5]	0.994
FEV_1_ (L)	0.36 ± 0.31 [(−0.36)‐1.35]	0.36 ± 0.38 [(−0.36)‐1.35]	0.33 ± 0.22 [(−0.22)‐0.70]	0.921
%FEV_1_ (%)	14.6 ± 12.3 [(−15.2)‐47.0]	14.4 ± 12.0 [(−15.2)‐43.4]	15.1 ± 13.6 [(−8.4)‐47.0]	0.853
%DLco (%)	19.7 ± 18.3 [(−11.0)‐121.4]	19.2 ± 11.8 [(−11.0)‐43.4]	20.7 ± 27.8 [1.3–121.4]	0.175
Gap between the predicted reduction and the actual volume				
VC (L)	0.01 ± 0.34 [(−1.05)‐0.84]	0.00 ± 0.34 [(−1.05)‐0.84]	0.03 ± 0.32 [(−0.63)‐0.57]	0.712
%VC (%)	1.2 ± 10.7 [(−28.1)‐26.6]	1.1 ± 10.1 [(−26.9)‐18.1]	1.8 ± 13.0 [(−28.1)‐26.6]	0.801
FEV_1_ (L)	0.08 ± 0.27 [(−0.42)‐0.87]	0.08 ± 0.25 [(−0.42)‐0.80]	0.08 ± 0.30 [(−0.38)‐0.87]	0.977
%FEV_1_ (%)	4.4 ± 11.0 [(−29.5)‐30.2]	4.3 ± 9.5 [(−13.4)‐28.2]	4.5 ± 15.4 [(−29.5)‐30.2]	0.945
%DLco (%)	0.32 ± 18.5 [(−98.4)‐32.6]	0.32 ± 12.1 [(−30.2)‐32.6]	0.31 ± 28.1 [(−98.4)‐24.6]	0.124

Abbreviations: DLco, diffusing capacity of the lung carbon monoxide; FEV_1_, forced expiratory volume in 1 second; SD, standard deviation; VC, vital capacity.

^a^ In bilateral patients, the postoperative pulmonary function was the volume calculated after the secondary surgery.

^b^ Comparisons between patients with ipsilateral and bilateral disease.

The gaps between the predicted and the actual reduction in the postoperative pulmonary function were as follows; mean ± SD and range: VC, 0.01 L ± 0.34 (range, −1.10 to 0.84 L); %VC, 1.2% ± 10.7 (range, −28.1% to 26.6%); FEV_1_, 0.08 L ± 0.27 (range, −0.42 to 0.87 L), %FEV_1_, 4.4% ± 11.0 (range, −29.5% to 30.2%); %DL_CO_, 0.32% ± 18.5 (range, −98.4% to 32.6%). Positive numbers mean that the actual reduction volume was less than the predicted volume.

There were no significant differences in these between the ipsilateral and bilateral patients.

### Histopathology, most advanced stage, and adjuvant therapy

The details of the histopathological findings, the most advanced stage, and adjuvant therapy are shown in Table 4. For histopathological types, 80 patients (94.1%) included adenocarcinoma and 37 patients (43.5%) included carcinoma in situ. The pathological stages of the most advanced lesions were stage 0–I in 58 patients (68.2%) and stage ≥II in 27 patients (31.8%). Lobectomy was performed in 23 patients and segmentectomy was performed in four patients. Adjuvant chemotherapy after surgery was performed in 36 patients (42.4%), with UFT in 22 patients and platinum combination therapy in 14 patients.

**TABLE 4:** Histopathology, most advanced stage, and adjuvant therapy in patients with synchronous multiple primary lung cancer

Variables	Total (*n* = 85)		Ipsilateral (*n* = 67)	Bilateral (*n* = 18)
	N	%	N	N
Histopathology				
Ad‐Ad	67	78.8%	55	12
Ad‐Sq	6	7.1%	5	1
Sq‐Sq	2	2.3%	1	1
Ad‐Ad‐Ad	3	3.5%	3	0
Ad‐Ad‐Sq	1	1.2%	0	1
Ad‐Ad‐Ad‐Ad	1	1.2%	0	1
Ad‐Ad‐Ad‐Sq	1	1.2%	0	1
Others	4	4.7%	3^a^	1^b^
Most advanced stage				
0	7	8.2%	6	1
IA1	15	17.7%	10	5
IA2	12	14.1%	11	1
IA3	8	9.4%	5	3
IB	16	18.9%	13	3
IIA	6	7.1%	6	0
IIB	7	8.2%	6	1
IIIA	10	11.8%	7	3
IIIB	2	2.3%	1	1
IVA	2	2.3%	2	0
Adjuvant therapy				
None	49	57.6%	38	11
Tegafur/uracil (UFT)	22	25.9%	18	4
Platinum combination therapy	14	16.5%	11	3

Abbreviations: Ad, adenocarcinoma; Sq, squamous cell carcinoma.

^a^ Adenocarcinoma and typical carcinoid (*n* = 1), adenosquamous carcinoma and squamous cell carcinoma (*n* = 2).

^b^ Squamous cell carcinoma and small cell carcinoma (*n* = 1).

### Postoperative complications

The postoperative complications are shown in Table 5. Postoperative complications were observed in 29 patients (34.1%); prolonged air leak for ≥7 days after surgery, which was observed in 15 patients, was the most common. Operative death within 30 days after surgery occurred in two patients (2.4%) (pneumonia, *n* = 1; cerebral infarction, *n* = 1). The incidence of postoperative complications in the ipsilateral and bilateral surgery groups did not differ to a statistically significant extent. No patients required home oxygen therapy after surgery.

**TABLE 5:** Postoperative complications in patients with synchronous multiple primary lung cancer

Variables	N (%)			*p* ^a^
	Total	Ipsilateral	Bilateral	
	(*N* = 85)	(*N* = 67)	(*N* = 18)	
Patients with complications	29 (34.1%)	26 (38.8%)	3 (16.7%)	0.066
Persistent air leak^b^	15	13 (19.4%)	2 (11.1%)	0.392
Pneumonia	3	2	1	
Delayed pulmonary fistula^c^	2	2	0	
Chylothorax	2	2	0	
Empyema	1	1	0	
Pleuritis	1	1	0	
Atelectasis	2	2	0	
Stress cardiomyopathy	1	0	1	
Recurrent laryngeal nerve palsy	1	1	0	
Cerebral infarction	1	1	0	
Surgical site infection	1	1	0	
Subcutaneous hematoma	1	1	0	
Subclavian artery injury	1	0	1	
Required home oxygen therapy	0	0	0	
Mortality	2 (2.4%)	1 (1.5%)	1 (5.6%)	0.362

^a^ Comparisons between patients with ipsilateral and bilateral disease.

^b^ Persistent air leak was defined as air leak that continued for >7 days after surgery.

^c^ Delayed pulmonary fistula was defined as air leak occurring after discharge.

### Prognosis

**FIGURE 2:**
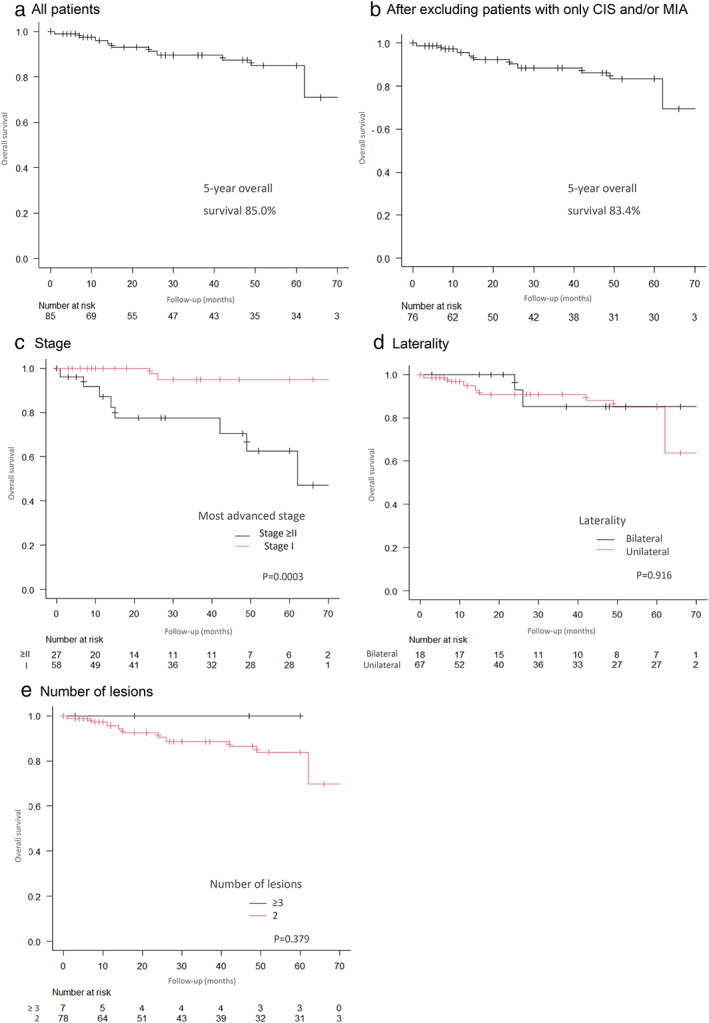
(a) Overall 5‐year survival curve in all patients (85.0%, *n* = 85). (b) Overall 5‐year survival curve after excluding patients with only carcinoma in situ and/or minimally invasive adenocarcinoma (83.4%, *n* = 76). (c) Five‐year survival curves according to the most advanced stage: stage I (*n* = 58), stage ≥II (*n* = 27). The 5‐year survival rate of patients with stage I disease was 94.9%, that of patients with stage ≥II disease was 62.6%; the difference was statistically significant (*p* < 0.001). (d) Five‐year survival curves according to laterality: bilateral (*n* = 18); ipsilateral (*n* = 67). The prognosis of patients with bilateral disease and those with ipsilateral disease did not differ to a statistically significant extent (85.1% vs. 85.0%, *p* = 0.916). (e) Five‐year survival curves according to the number of lesions: two lesions (*n* = 78); three or four lesions (*n* = 7). The prognosis of patients with two lesions and that of patients with three or four lesions did not differ to a statistically significant extent (83.8% vs. 100%, *p* = 0.379)

Recurrence was observed in 20 patients (23.5%); 14 had pathological stage ≥II, 11 had lymph node metastasis, and two had pleural dissemination. The patterns of recurrence included metastasis to other organs, pleural dissemination, and lymph node metastasis; no local recurrence was observed. In the recurrent patients, the surgical procedure for the most advanced lesion was lobectomy in 16 patients and segmentectomy in four patients. The pattern of recurrence in patients who received segmentectomy was distant metastasis in two patients and pleural dissemination in two patients; no stump recurrence or lymph node recurrence was observed. The mean recurrence‐free survival period was 32.5 months, while the 5‐year survival rate was 85.0% (Figure 2(a)). After excluding patients with only carcinoma in situ and/or minimally invasive adenocarcinoma, the 5‐year survival rate was 83.4% (Figure 2(b)). The 5‐year survival rate according to the most advanced pathological stage was 94.9% in patients with stage I disease and 62.6% in those with stage ≥II disease; the difference was statistically significant (*p* < 0.001, Figure 2(c)). In contrast, the 5‐year survival rate did not differ according to laterality or the number of lesions (Figure 2(d),(e)).

## DISCUSSION

Recently, with the advent of high‐resolution chest imaging and lung cancer screening programs, the number of patients diagnosed with MPLC is increasing.^1^ Since most patients show ground‐glass opacities (GGO),^15^, ^16^ treatment strategies that include surgery will be considered; however, at present, there are no criteria for the surgical management of MPLC. Depending on the number and localization of tumors, an appropriate surgical procedure that preserves the pulmonary function while ensuring curability is required.

Multiple lung cancer includes SMPLC, metastatic multiple lung cancer, metastatic lung tumor, and other conditions; thus, the differential diagnosis is important for determining the best treatment strategy.^9^ In our department, the treatment strategy for SMPLC is based on the principle of complete resection of all lesions except pure GGO lesions of ≤2 cm. In MPLC, there has been a transition from reports^5^, ^17^, ^18^, ^19^ that performed lobectomy and pneumonectomy to reports^6^, ^7^, ^8^ that recommend limited resection, including segmental and wedge resection. Conventionally, a predicted postoperative VC of ≥1000 ml, a predicted postoperative FEV_1_ of ≥800 ml, and a predicted postoperative %FEV_1_ was >40% are often the standard conditions for performing lung resection; however, in recent years, the DL_CO_ is also widely recognized as an important index, and it is considered that the risk of perioperative mortality increases when the postoperative predicted %DL_CO_ is ≤40%.^11^, ^13^ Our department has also established surgical indications based on these factors, and %DL_CO_ was also examined in 81 patients (95.3%).

In this study, 76 patients (89.4%) underwent surgery combined with limited resection (e.g., segmentectomy and wedge resection), and the proportion of limited resection was higher in comparison to previous reports.^5^, ^7^, ^19^ On the other hand, nine patients (10.6%) underwent resection of two lobes (upper‐middle lobectomy [*n* = 5], middle‐lower lobectomy [*n* = 3], middle lobectomy, and left lower lobectomy [*n* = 1]), all of which included middle lobectomy. Resection of two lobes, including bilobectomy, has been reported to increase postoperative cardiopulmonary complications,^20^, ^21^ and limited resection should be selected as much as possible unless the small‐volume middle lobe is involved. For good indications for limited resection, physicians should refer to the results of comparative trials between lobectomy and limited resection for lung cancer with a maximum tumor diameter of ≤2.0 cm (JCOG0804/WJOG4507L^22^ and JCOG0802/WJOG4607L, which will be finally reported from Japan after 2021.). However, in patients of SMPLC, it is often necessary to select passive limited resection due to the patient's poor cardiopulmonary function, and the results of clinical trials cannot be generally applied. It is important to select a surgical procedure with the overall assessment of the total size, consolidation diameter, number and localization of tumors, pulmonary function, and the SUVmax on FDG‐PET.^23^, ^24^, ^25^ However, if the patient's cardiopulmonary function is poor and surgery is not indicated, other treatments, such as radiation therapy, should be considered.

We examined the postoperative pulmonary function of patients with SMPLC in detail. To our knowledge, this is the first study to compare the predicted pulmonary function and actual pulmonary function after surgery in patients with SMPLC. The predicted volume reduction after surgery was calculated based on the number of subsegmental bronchi to be resected; however, there was no serious discrepancy between the predicted volume reduction and the actual volume reduction after surgery. In addition, no patients required home oxygen therapy due to unexpected pulmonary dysfunction after surgery. Accordingly, we consider that the prediction by the calculation method that we applied was accurate and very useful for selecting an appropriate surgical procedure for SMPLC.

Regarding the prognosis of SMPLC, although a certain number of carcinomas in situ were included, the 5‐year survival rate after surgery was 85.0% in our study, which was superior to the rates in previous reports (34.0%–70.3%).^3^, ^4^, ^5^, ^9^, ^18^, ^26^ In addition, the 5‐year survival rate according to the most advanced pathological stage was the same as or better than the data put out by the International Association for the Study of Lung Cancer (IASLC).^27^ Therefore, a good long‐term survival of SMPLC comparable to that with solitary cancers was considered achievable with surgery. Patients of SMPLC are diverse and it is often difficult to select a treatment strategy; however, a good prognosis can be obtained by selecting an appropriate surgical procedure for the individual patient. Thus, the role of surgery, including limited resection, is considered important.

The present study was associated with some limitations. First, it was retrospective in nature and performed in a single‐center. Second, the number of patients, especially those involving bilateral SMPLC, was small. Finally, the rate of reduction of the pulmonary function in wedge resection was treated as 2% in all patients; however, only slight differences were found in each patient. This method was applied based on our hypothesis that wedge resection can be considered to be almost equivalent to one subsegmental resection. We then found that it was a valid evaluation because there was no serious discrepancy between the predicted volume reduction and the actual volume reduction after the surgery in patients that involved wedge resection.

In conclusion, we examined the surgical treatment of SMPLC, including the pre‐ and postoperative pulmonary function. To our knowledge, this is the first study to compare the predicted pulmonary function and actual pulmonary function after surgery in SMPLC. Our method could adequately predict postoperative pulmonary function, and the prognosis was good when combined with limited resection. Taken together, we consider that surgical treatment, including limited resection, for SMPLC is a potentially curative treatment that has the potential to preserve pulmonary function.

## CONFLICT OF INTEREST

All authors declare no conflicts of interest in association with the present study.
